# Effects of Slaughter Positions on Catecholamine, Blood Biochemical and Electroencephalogram Changes in Cattle Restrained Using a Modified Mark IV Box

**DOI:** 10.3390/ani11071979

**Published:** 2021-07-01

**Authors:** Jurhamid Columbres Imlan, Ubedullah Kaka, Yong-Meng Goh, Zulkifli Idrus, Elmutaz Atta Awad, Ahmed Abubakar Abubakar, Tanbir Ahmad, Hassan N. Quaza Nizamuddin, Awis Qurni Sazili

**Affiliations:** 1 Institute of Tropical Agriculture and Food Security, Universiti Putra Malaysia, Serdang 43400, Selangor, Malaysia; jurhamidimlan@yahoo.com.ph (J.C.I.); dr_ubedkaka@upm.edu.my (U.K.); ymgoh@upm.edu.my (Y.-M.G.); zulidrus@upm.edu.my (Z.I.); atta.am@umk.edu.my (E.A.A.); ahmadsadeeq7@gmail.com (A.A.A.); 2 Department of Animal Science, College of Agriculture, University of Southern Mindanao, Cotabato 9407, Philippines; 3 Department of Companion Animal Medicine and Surgery, Faculty of Veterinary Medicine, Universiti Putra Malaysia, Serdang 43400, Selangor, Malaysia; 4 Department of Preclinical Sciences, Faculty of Veterinary Medicine, Universiti Putra Malaysia, Serdang 43400, Selangor, Malaysia; 5 Department of Animal Science, Faculty of Agriculture, Universiti Putra Malaysia, Serdang 43400, Selangor, Malaysia; tanbirve05@gmail.com; 6 Preclinical Department, Universiti Malaysia Kelantan, Pengkalan Chepa 16100, Kelantan, Malaysia; 7 ICAR—Indian Veterinary Research Institute (IVRI), Izatnagar, Bareilly 243122, India; 8 Department of Veterinary Services, Wisma Tani, Blok Podium, Putrajaya 62630, Selangor, Malaysia; Hassan@dvs.gov.my; 9 Halal Products Research Institute, Universiti Putra Malaysia, Putra Infoport, Serdang 43400, Selangor, Malaysia

**Keywords:** Halal slaughter positions, electroencephalographic response, blood biochemical, catecholamines, animal welfare, cattle

## Abstract

**Simple Summary:**

Slaughter position plays a crucial role from an animal welfare perspective (fear due to novelty, discomfort, and pain), as it involves handling by workers and restraint methods at abattoirs. In ritual slaughter, it is obligatory to restrain the animal in a prescribed position (lateral or upright), and lateral recumbency in Halal slaughter takes minimum effort in well-equipped abattoirs with trained staff compared to less equipped abattoirs with less trained personnel or, worst of all, outside of abattoirs. Halal slaughter is a technique practiced around the world by Muslims. No studies have been reported comparing upright and lateral positions in Halal slaughter and their impact on cattle blood biochemistry and pain sensation. Therefore, this study was designed to evaluate responses in cattle subjected to upright and lateral slaughter positions on EEG and catecholamines. The results revealed that the two positions had different effects on brain activity and blood parameters.

**Abstract:**

The proper slaughter positioning of animals is among the most crucial factors in animal welfare. The lateral position in Halal slaughter is a technique used around the world by Muslims, with a few practicing the upright position. The literature on the effects of slaughter in upright versus lateral positions on pain and stress is scarce. Thus, this study was designed to evaluate the effects of slaughter positions on blood biochemical parameters, plasma catecholamines, and electroencephalographic (EEG) responses. Twenty Brahman crossbred steers were subjected to slaughter in either lateral recumbency (LP) (*n* = 10) or an upright position (UP) (*n* = 10). There was a significant increase in adrenaline (*p* < 0.0001) and noradrenaline (*p* < 0.05) at T2 compared to T1 in the animals of both groups. A significant difference (*p* < 0.0001) was observed in the median frequency (MF) and total power (Ptot) of EEG, parameters for pain and stress, between the animals slaughtered in the upright and the lateral position. However, MF and delta waves were significantly higher (*p* < 0.05) after slaughter in the UP group than in the LP group. The results demonstrate a lesser amount of stress and pain responses among the LP group.

## 1. Introduction

Globally, the demand for meat, which brings about the need for animals to be slaughtered, has always been high and has only increased in recent decades; as such, the handling and management of these animals during slaughter has long been a source of contention from a welfare standpoint. The Halal slaughter method is an obligatory practice by Muslims. The Halal and kosher slaughter methods are also used commercially on a larger scale to meet the global volume of ritually slaughtered meat [1]. It is thought that religious slaughter conducted properly and in accordance with guidelines improves animal welfare [2]. Indeed, previous research has shown that when cattle are slaughtered using upright restraint or cast, the ends of the severed carotid arteries develop a false aneurysm [1]. By contrast, improperly trained slaughterman and undesirable restraint systems in some commercial production facilities cause unnecessary animal suffering. Currently, concerns regarding religious slaughter are centered around stress due to pre-slaughter handling, restraint methods, the pain and distress that may be felt during and after neck cut, and the prolonged duration to loss of brain function and death if stunning is not applied [3].

The use of restraints to hold animals in place for easy handling and slaughtering, the welfare of the slaughtered animals, and the workers is considered an essential pre-slaughter practice. There is a scarcity of scientific information on the effect of the commonly-used restraining methods in ritual slaughter (Halal) on cattle welfare, especially the impact of positioning and rotation of the animal. The majority of Halal animals are slaughtered in left lateral recumbency, with a few countries practicing the upright slaughter position—for instance, Canada [4]. By contrast, inverting fully conscious animals for ritual slaughter can cause considerable distress and fear in all animals due to the aspiration of rumen fluids (ruminants) and the compression of internal thoracic organs inhibiting respiration, especially in adult bovines, steers, and heavy calves [4]. Research has been conducted on animals slaughtered in various positions such as upright, 45°, 90° or lateral recumbency, and 180° [5,6,7,8].

For the proper conduct of Halal slaughter, and especially non-stunning Halal slaughter, it is essential to restrain animals properly. In the Halal slaughter method, at the time of neck cut, animals are laid on their left flank facing the Qibla (direction of Makkah, a sacred site in Saudi Arabia). Thus, the animal’s face and the slaughterer should face the Qibla [9]. This position’s main objective is to drain the maximum amount of blood through the body’s pressure on the heart [10], as the consumption of blood is forbidden to Muslims (Quran 2:173, 5:3, 6:145, 16:115). As prescribed in the hadith, the animal should be shackled and elevated only once unconscious and bleeding [11]. Some researchers have supported a horizontal position rather than vertical hanging during the slaughter process to achieve greater blood loss [12,13]. Modern slaughterhouses have used various restraining methods, such as inverting animals on their backs, lateral recumbency, an upright or standing position, and hoisting conscious cattle by the hind legs [14,15]. The hoisting of fully conscious cattle, which is practiced in some countries, causes unnecessary suffering and pain due to their heavy weight [16]. This inhumane restraint is against Islamic teachings, EU legislation, and animal welfare guidelines. The American Society for the Prevention of Cruelty to Animals (ASPCA) box is used to restrain animals in the upright position with a chin lift that stretches the neck to provide easy access for Halal incision [14]. However, there is the possibility of aspiration of blood into the lungs or poor bleeding due to the clamping of the blood vessels of the neck against the head restraint, resulting in a delayed loss of consciousness [6]. Although not preferred by individual Muslims, upright restraint has been widely used by European Halal authorities [5]. The most recommended Halal slaughter method is the lateral recumbency position (at a 90° angle, lying on the side) due to its compliance with Halal criteria [14]. In lateral recumbency, cattle do not suffer from pressure on the diaphragm, aorta, or major veins; however, pressure on other internal organs may occur [17]. Large animal slaughterhouses use a V-shaped or straddled conveyor with a full or half inversion in a rotary pen and an upright restraint system [15].

Velarde et al. [8] evaluated various positions for animal restraint, such as turning by 45°, 90° (on their sides), 180° (on their back), and upright during Halal slaughter [8]. They reported more struggling and vocalization and increased bleeding efficiency in cattle turned on their sides compared to those slaughtered in an upright position. Fewer cuts were performed on cattle restrained on 90° (three cuts) compared to those on 180° (five cuts) and in an upright position (nine cuts) [8]. Aspiration of blood into the respiratory tract has been reported in cattle during slaughter in the upright [5] and inverted positions [18]. No study has been conducted on animals slaughtered following the lateral and upright positions in Halal, comparing the effects of these two slaughter methods on the physiological indicators and animal welfare.

For the advancement of animal welfare, the degree of pain and distress that animals experience as a result of slaughter must be continuously studied. During slaughter, pain reactions in animals may be concealed by the restraining objects or the shackling tool [16]. Electroencephalograms (EEGs) have thus been widely used to assess animals’ responses to pain. There is a significant correlation between EEG variables and subjective pain evaluation or animal welfare [19]. The moment animals suffer pain, they exhibit behavioral changes and produce EEG spectral changes that indicate distress [20].

It was hypothesized that the lateral slaughter position would produce less stress and pain than the upright slaughter position, as exhibited in the blood biochemistry and electroencephalograms in cattle subjected to restraint using the modified Mark IV box. Therefore, this study aimed to compare the effects of lateral versus upright slaughter positions on EEG changes, catecholamines, and blood biochemical parameters in cattle subjected to restraint using the modified Mark IV box.

## 2. Materials and Methods

### 2.1. Animals

**Figure 1:**
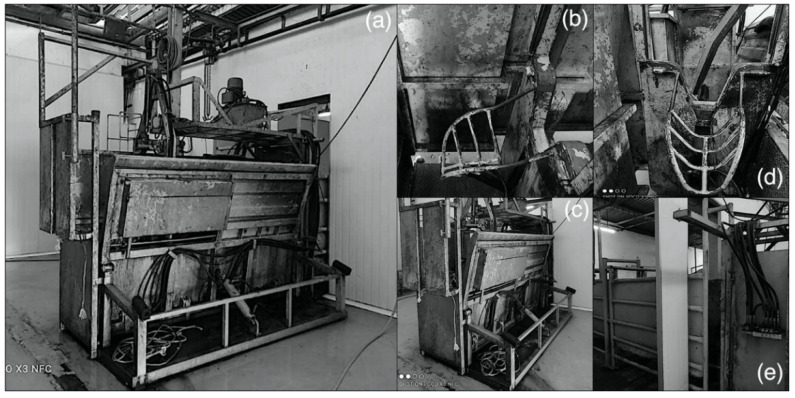
(**a**)-Modified MARK IV restraint box. (**b**)-chin lift, (**c**)-the box showing the part where animal is turned on its lateral side, (**d**)-chin lifter and clamp to hold animal while turning on lateral recumbency, (**e**)-side view.

This study was conducted following the animal ethics guidelines of the Research Policy of Universiti Putra Malaysia (UPM/IACUC/R028/2016). A total number of 20 Brahman crossbreed steers, with a live weight of about 420.00 ± 20.0 kg, were obtained from Katherine, a town situated in Australia’s Northern Territory. The animals were transported via sea (for 14 d) from Darwin Port Australia to Pasir Gudang Port Johor, Malaysia. After that, the animals were road transported from Pasir Gudang Port to Universiti Putra Malaysia (UPM) Serdang, Selangor. Animals were fattened for five months at the animal facility of the Institute of Tropical Agriculture and Food Security (ITAFoS) before road transport (30 km) to the Ruminant Commercial Abattoir, Department of Veterinary Services, Shah Alam, Selangor, for slaughter. The cattle were assigned to two groups based on slaughter positions, the lateral recumbency position (LP) group (*n* = 10) and the upright position (UP) group (*n* = 10). In the current study, a modified Mark IV box was used (operated entirely on a hydraulic system and fitted with a chin lift) to ease handling and restrain animals. The modified Mark IV box can restrain animals from movement with ease and comfort without any risk of injury to either the animal or the operators. The modified Mark IV restraint box is fitted with an adjustable chin lift, making it easier to hold the head and adjust the animal’s position, which gives room for upright slaughter with ease. Additionally, the box can turn on the lateral (90°) side at the operator’s will. All the animals were slaughtered using a modified MARK IV restraint box (Figure 1a–e). The slaughtering of animals was performed at the Ruminant Commercial Abattoir, Department of Veterinary Services, Shah Alam, Selangor, Malaysia. The slaughtering was done in a Halal manner, as illustrated in MS1500: 2009 [21]. This procedure entails cutting the carotid supply routes, jugular veins, trachea, and throat. Blood parameters and electroencephalography data were acquired pre-slaughter (T1) and post-slaughter (T2) after the neck cut. Neck cutting was carried out at the 1st cervical (C1) vertebra [22]. Before the animals were slaughtered, they were restricted from feed for 3 h and provided drinking water ad libitum.

### 2.2. Electroencephalography

Electroencephalogram activity for individual animals was recorded before neck cut (i.e., pre-slaughter (T1)) and after neck cut (T2) for about 7 min using a Power Lab Bio Potential Recordings system device (Power Lab data acquisition system, AD Instruments Ltd., Sydney, Australia). Upon entry to the restraint box, the animal was allowed to relax for a few seconds, the baseline blood sample was taken, and then two Kendall (Covidien 11c, 15 Hampshire Street, Mansfield 02048, MA, USA) conductive adhesive hydrogel foam electrodes were placed 6–8 cm distally from the poll at an equal distance from the anterior orbital prominences of both the left and right eyes and on the left base of the poll. The EEG recordings were acquired within a band-pass signal range between 0.1 and 200 Hz, at a sampling rate of 1 kHz. These signals were then analyzed offline with the help of the Chart Spectral Analysis function of Chart 5.0 (Powerlab.data acquisition system, Sydney, Australia). Prior to EEG analysis, the raw EEG recordings were resampled at 1024 Hz, and only frequencies between 0.1 and 30 Hz were obtained, to minimize the presence of artefacts. Possible interferences from concurrent electrocardiography signals were digitally removed from the raw EEG recordings using Chart 5.0 software (AD Instruments) before analysis. The signals were then processed in blocks of 1 s epochs, yielding 60 epochs per minute. The signal was then filtered into band-pass filters to yield delta (0.1–4 Hz), theta (4.1–8 Hz), alpha (8.1–12 Hz), and beta (12.1–20 Hz) waves. The Chart Spectral Analysis Function (Chart 5.0 software, AD Instruments, Sydney, Australia) was used to analyze each frequency component. Briefly, the signals were subjected to fast Fourier transformation (FFT), and power–density curves for each frequency band were derived on the basis of cosine bell distribution. Each calculation of the alpha, beta, delta, and theta waves was done for the pre-slaughter and post-slaughter root mean square (RMS). Median frequency (F50; the frequency below 50% of the total power of the EEG) and total power (Ptot; the total area under the power spectrum curve) pre-slaughter and post-slaughter were also determined.

### 2.3. Blood Sampling

Blood was collected via jugular venipuncture pre-slaughter in the restraint box for T1, and from blood flow for T2 (post-neck-cut). Blood samples for the biochemical analyses were collected into a vacutainer (BD Franklin Lakes, NJ, USA), stored in ice, and taken to the Clinical Pathology and Hematology Laboratory, Faculty of Veterinary Medicine, Universiti Putra Malaysia, after about one hour. The blood samples required for catecholamines (adrenaline and noradrenaline) or hormonal analyses were collected into vacutainer K3 ethylene diamine tetra acetic acid (EDTA) tubes and slanted in crushed ice, after which the samples were centrifuged at 800 rpm and 4 °C for 15 min. The retrieved plasma portion was separated into 2 mL aliquots and kept at −80 °C until analysis.

### 2.4. Determination of Blood Biochemical Parameters

Lactate, glucose, urea, total protein, creatinine, creatine kinase (CK), calcium, and lactate dehydrogenase (LDH) were determined using a chemistry analyzer (Auto Analyzer Hitachi 902, Tokyo, Japan).

### 2.5. Determination of Adrenaline

The adrenaline (epinephrine) content in the blood was analyzed quantitatively with the use of an Adrenaline Plasma Enzyme-Linked Immuno Sorbent Assay (ELISA) High Sensitive kit # BA E-4100 (LDN^®^, Nordhorn, Germany). The competitive ELISA kit uses the microtiter plate format. Adrenaline was extracted from a plasma sample with the help of a cis-diol-specific affinity gel, acylated, and then modified enzymatically. The antigen was bound to the solid phase of the microtiter plate, and the derivatized standards, controls, and samples, as well as the solid phase bound analytes, competed for a fixed number of anti-serum binding sites.

#### 2.5.1. Sample Preparation, Extraction and Acylation

A total of 25 µL of standards and controls, as well as 400 µL of plasma samples were pipetted into the respective wells of the extraction plate. A total of 500 µL of deionized water was added to the wells with the standards and the controls, while 200 µL of deionized water was added to the wells with the samples. The contents were mixed by shaking the plate on a microplate shaker for 1 min. Thereafter, 25 µL of Tris EDTA buffer was pipetted into all wells, and the plate was covered with adhesive foil and incubated at room temperature (20–25 °C) for 60 min on a plate shaker (MS Major Science, Taoyuan, Taiwan) at 600 rpm. Following incubation, the foil was removed, and the plate was blot-dried by inverting it and tapping it onto a clean, lint-free towel. A total of 1 mL of wash buffer was pipetted into all wells, and the plate was shaken at 600 rpm on a plate shaker for 5 min at room temperature. The plate was then blot-dried by turning it over and tapping on a neat tissue towel. The washing was repeated one time. After washing, 150 µL of acylation buffer followed by 25 µL of acylation reagent were pipetted into all wells. In order to ensure rapid addition, an 8-channel pipette (Biopette ATM, Limbowa Gdansk, Poland) was used. Incubation of the plate for 20 min was carried out at room temperature on a plate shaker at 600 rpm. The content in the plate was then evacuated completely and blot-dried by turning over and tapping on a clean lint-free towel. A total of 1 mL of wash buffer was pipetted into all wells, and the plate was shaken for 5 min at room temperature. Blot drying of the plate was conducted by turning the plate over and tapping it on a neat tissue towel. Washing was then repeated. Following washing, 100 µL of HCl was pipetted into all wells using an 8-channel pipette. A foil was used to cover the plate, and the plate was incubated on a plate shaker at 600 rpm for 10 min at room temperature.

#### 2.5.2. Enzymatic Conversion

Using the above contents of the extraction plate, 90 µL of each of the extracted standards, control, and samples were pipetted into the various wells of the microtiter plate. Using an 8-channel pipette, 25 µL of the enzyme solution was added to all wells. The plate was then shaken for 1 min on a shaking incubator after being covered with foil. Lastly, the plate was incubated (Shaker Incubator, Hotech^®^ 702R, Taipei, Taiwan) for 2 h at 37 °C.

#### 2.5.3. Adrenaline Evaluation

Using the contents of the enzyme plate above (100 µL of standards), the control and samples were pipetted into the respective pre-coated Adrenaline Mikrotiter Strips (LDN^®^, Nordhorn, Germany). Using an 8-channel pipette, 50 µL of the respective Adrenaline antiserum was added to all wells. At room temperature, the plate was incubated for 1 min on a shaker at 600 rpm after being covered with foil. This was followed by incubation of the plate overnight (15–20 h) at 4 °C. The next day, the foil was removed, and the contents of the plate were discarded. Each well was thoroughly washed with 300 µL wash buffer 4 times and then blot-dried by tapping and turning over the plate on clean tissue towels. Thereafter, 100 µL of substrate was included in all wells. Incubation of the plate was then carried out at room temperature for 25 min on a plate shaker at 600 rpm while preventing exposure to sunrays. Finally, 100 µL of stop solution was pipetted into all the wells, and the absorbance was read within 10 min with the use of an auto UV Xenon flash lamp micro plate reader (infinite M200, Tecan, Austria) set to 450 nm, with a reference wavelength between 620 nm and 650 nm. Absorbance readings of the standards (linear, y-axis) were plotted against the corresponding standard concentrations (logarithmic, x-axis) to obtain the calibration curve. A non-linear regression (4-parameter) was adopted for curve fitting. The plasma sample concentration was obtained from the standard curve, which was then multiplied by the volume factor to compensate for dilution during extraction. The volume factor was derived from the following equation:

Volume factor = (600 µL)/(used plasma volume (µL))

#### 2.5.4. Determination of Noradrenaline

The quantitative analysis of the noradrenaline (norepinephrine) content in the blood was carried out using a Noradrenaline Plasma ELISA High Sensitive kit # BA E-4200 (LDN^®^, Nordhorn, Germany). This kit works under the same principle as that explained above for adrenaline. The sample preparation, extraction, acylation, and enzymatic conversion were carried out following a procedure similar to the one discussed above. Ten microliters of the standards, control, and samples were pipetted from the plate of the enzyme into the individual pre-coated Noradrenaline Microtiter Strips. Using an 8-channel pipette, 50 µL of the Noradrenaline antiserum was added to all the wells. The steps that followed (i.e., covering with foil through to the calculation of noradrenaline concentration) were similar to those described in the section for adrenaline.

#### 2.5.5. Statistical Analysis

Before analysis, data were checked for their conformance to the normal distribution using a Shapiro–Wilk test. The analysis was carried out using a 2 × 2 factorial design with sampling points and slaughter positions designated as factors. Statistical analysis was performed using the General Linear Model (GLM) procedure of the Statistical Analysis System (SAS) package Version 9.4 (SAS Institute Inc., Cary, NC, USA) 2007 [23]. Data analysis was carried out with interactions using the sampling points and slaughter positions as the main effects, using the repeated measure analysis of variance (RM-ANOVA) procedure. Duncan’s multiple-range test was used to distinguish significantly different means. Statistical significance was set at *p* < 0.05.

## 3. Results

### 3.1. Blood Biochemical Parameters

The results of the biochemical parameters of this study are shown in Table 1. At T1, the upright slaughter position resulted in higher (*p* < 0.05) glucose levels when compared to the lateral position. Similarly, higher (*p* < 0.05) glucose levels were recorded in the upright slaughter group than those obtained for the lateral group at T2. Likewise, the upright slaughter position resulted in higher (*p* < 0.05) creatine kinase levels compared to those obtained from animals subjected to the lateral slaughter position at T1. Similarly, higher (*p* < 0.05) levels of creatine kinase were also recorded in animals slaughtered in an upright position than those obtained for the lateral position at T2.

In comparison to the lateral position, subjecting animals to the upright position also resulted in an increased level (*p* < 0.05) of lactate dehydrogenase in the animals at T2. At post-slaughter, the lactate dehydrogenase levels remained higher (*p* < 0.05) in the upright group than in the lateral group. Additionally, calcium levels in animals subjected to the lateral slaughter position and upright slaughter positions showed no pre-slaughter differences. Similarly, there was no significant difference in calcium levels for animals subjected to lateral and upright slaughter positions post-slaughter. A similar trend was observed for total protein and creatinine, where no significant differences were observed at T1 and T2 for both slaughter restraining positions (lateral and upright). However, lactate levels were significantly higher in animals subjected to the upright slaughter position at T1. No significant difference in lactate levels was seen for animals subjected to either the upright or lateral positions at T2.

### 3.2. Influence of Slaughter Positions on Hormonal Parameters 

The concentration of adrenaline affected by slaughter positions and sampling points in this study is shown in Table 2. There was a significant interaction (*p* < 0.0001) between the sampling position and sampling points for adrenaline. Higher adrenaline concentrations (*p* < 0.0001) were recorded in samples obtained at T2 than in samples collected at T1 in animals subjected to both the lateral and upright slaughter positions. No significant interaction was observed (*p* = 0.507) between slaughter positions, and sampling periods were noted for noradrenaline concentration. The noradrenaline concentration was significantly higher in animals slaughtered in an upright position than in a lateral position. These were observed at both T1 and T2 (*p* < 0.0001). Additionally, the concentrations of noradrenaline were significantly elevated at T2 compared to T1, and this was found in animals slaughtered in both the lateral and upright positions (*p* < 0.05).

### 3.3. Influence of Slaughter Positions on EEG Recording

Table 3 shows the electroencephalographic changes in animals subjected to different slaughter positions and sampling points. No significant interactions (*p* > 0.05) between slaughtering positions and sampling time points were seen for alpha, beta, Ptot, and MF. Regardless of treatment, alpha, beta, delta, theta, Ptot, and MF (F50) were significantly higher (*p* < 0.0001) at T2 than at T1. Irrespective of the time points, MF was markedly higher in the upright, over the lateral, position, whereas no significant difference was recorded between treatments for alpha (*p* = 0.735), beta (*p* = 0.249), delta (*p* = 0.075), theta (*p* = 0.288), and Ptot (*p* = 0.178). On the other hand, there was a significant interaction (*p* < 0.05) between slaughtering positions and sampling time points for delta and theta waves. There were significantly higher values at post-slaughter for animals in the upright position compared to animals in the lateral slaughter position for delta waves.

## 4. Discussion

### 4.1. Blood Biochemical Parameters

Before discussing the results of the current study, it is worth mentioning that, because of different technical constraints related to the upright and lateral slaughter positions in animals, blood samples were collected at two different points (T1 and T2). The values obtained at T1 may have been affected by many factors, such as noise, novel environment, unpleasant human contact, unfamiliar animals, and/or the act of restraining itself. Although animal’s slaughtered in an upright position were seen to be struggling upon neck cut which could likely be due to discomfort experienced during restraint. According to Grandin [6], intermittent noise can agitate animals, such as air hissing from pneumatic pipes, people or machines moving quickly, and metal objects banging. However, variations in values between the upright and lateral slaughter positions in animals suggest the occurrence of stress due to restraint in various positions in the current research. This is because, with the exception of the slaughter positions, all animals (or both groups) were subjected to similar conditions.

Generally, the slaughter positions affected the plasma stress parameters and selected frequency bands of the electroencephalogram. A useful diagnostic tool to determine the level and type of stress that animals experience during stunning and/or exsanguination is through exsanguinated blood [24,25,26]. Grandin [6] and Shaw [27] reported that short-term stresses increased glucose and lactate levels, even though the values of both appeared to be higher following an unpleasant handling situation. Glucose concentration increases through increased glycogenolysis and gluconeogenesis rates associated with increased catecholamine and the glucocorticoids released during stressful conditions [25,26,27,28]. Our study results agree with those of Gruber et al. [29]. The higher lactate content could be due to the physical movements (struggling, discomfort behavior) linked to animal restraint in a lateral position. These findings, combined with the results from [26,27], demonstrate that excitable animals mobilized more glucose through glycogenolysis due to increased energy demands in response to stress in the muscle than their less agitated counterparts, resulting in elevated lactate and glucose levels being transported into the blood. In farm animals, increased sympathoadrenal activity stimulated by physical and psychological stress leads to hyperglycemia due to the increased breakdown of glycogen in the liver [22,24,29]. This observation is in tandem with Grandin [22], who showed that the slaughtering of cattle without stunning causes blood lactate to increase due to fast anaerobic glycolysis. Animals that underwent normal pre-slaughter handling were found to have a lactate level that was slightly lower [30,31]. It has been reported that better pork quality is due to low lactate levels in the pig of origin during bleeding [32,33,34]. As observed in the present study, serum lactate was affected by the slaughter position only at pre-slaughter. Lower levels of lactate were found in animals subjected to the lateral slaughter position than their upright-position counterparts. The release of lactate is related to the physical stress experienced by the animals [35].

Moreover, Lima et al. [36] concluded that serum lactate in Nellore steers could be used as an evaluation tool for quality of handling. Peres et al. [37] related higher serum lactate concentrations to stress in pigs at slaughter. Additionally, [27] found that animals that reacted adversely to handling and chute restraint had more elevated plasma lactate at slaughter. Creatine kinase (CK) is an enzyme that is highly sensitive when less relevant activities are carried out. It speeds up creatine conversion to phosphocreatine, which is used to store energy in tissues. The increment in blood CK level shows how badly animals were handled before slaughtering and the rate at which the muscles were damaged during handling [17,36,37]. This enzyme is mostly situated in various tissues, and its appearance in the blood plasma serves as a sign of muscle damage [38]. Lactate dehydrogenase (LDH) is an enzyme that reacts to factors caused by stress or wearing of muscle. Creatine kinase and LDH are both enzymes released into the blood during muscle injury, as a sign of stress and fatigue [39]. In this study, CK and LDH levels were affected by the slaughter position; this was observed at both T1 and T2. Creatine kinase levels were numerically higher in the blood samples collected post-slaughter when animals were subjected to the lateral slaughter position. Meanwhile, irrespective of the slaughter position, the levels of LDH were only numerically higher in the post-slaughter samples. Pre-slaughter stress causes a rapid release of enzymes such as creatine kinase and lactate dehydrogenase into the blood, which depletes the glycogen storage in the body, triggering a lower rate of post-mortem lactic acid synthesis, high ultimate pH, undesirable color, and greater water-holding capacity [40].

Glucose levels in the blood were higher (*p* < 0.05) in upright position slaughter than in the lateral position. However, this result did not indicate a higher stress response in the upright group since the glucose values were within the normal range. Cardona and Mota-Rojas [41] mentioned that animals are exposed to new experiences that inevitably cause fear during slaughter, which causes evident physical and emotional stress. In the present study, the blood lactate concentration was significantly higher for the upright position than in the lateral slaughter position. Grandin [29] revealed that slaughtering cattle without prior stunning leads to a positive increase in blood lactate caused by fast anaerobic glycolysis. Regardless of slaughter position, the mean changes in glucose concentration and lactate were higher at post-slaughter when compared to pre-slaughter. Glucose concentration rises through increased glycogenolysis and gluconeogenesis rates due to the additional increment in catecholamine and glucocorticoids [42].

In this study, the levels of CK and LDH activity post-slaughter were higher than the pre-slaughter values for both the upright and lateral slaughter positions. These results are in line with the findings of [43] in goats and [44] in lambs, in which significant differences in LDH and CK activities were found between pre- and post-slaughter. Therefore, the present data highlight that physical restraint led to a change in the blood biochemical parameters. Thus, regardless of slaughter position, the act of slaughter may inflict a significant degree of changes on most of the blood biochemical parameters.

### 4.2. Influence of Slaughter Positions on Catecholamines

The response to acute trauma (such as that induced by slaughter position and neck cut) might lead to some emotional reactions that activate the nervous sympathetic–adrenomedullary system, which in turn responds to stress that lasts for a short period of time by secreting catecholamines [45].

While slaughtering animals in the upright position resulted in a two-fold increase in adrenaline concentration post-slaughter, the increase in adrenaline concentration post-slaughter was smaller among animals slaughtered in the lateral position. This indicates that epinephrine is the catecholamine released most often and in a large amount in situations that involve a fight or flight reaction of the Autonomic Nervous System (ANS) [46].

At the restraint and bleeding stage, the vast majority of the released noradrenaline in circulation emerged from sympathetic nerve endings instead of the adrenal medulla [47]. It is well known that both the sympathetic and the hypothalamic–pituitary–adrenal axes are involved when animals are exposed to stressful situations [48]. The activation of the sympathetic axis responds to short-term stress through the release of catecholamines (epinephrine and norepinephrine) into the bloodstream [3,21,49,50]. In our study, the higher level of catecholamines in animals slaughtered in the upright position compared to that of animals slaughtered in the lateral position indicates that the animals slaughtered in the upright position were more stressed than those in the lateral position. Catecholamines (dopamine, norepinephrine, and epinephrine) are vital neurotransmitters of the sympathetic nervous system [51]. Under reasonable physiological situations, catecholamines are discharged from the adrenal medulla to keep up with the homeostasis of the body and to adjust various functions of the body, which includes maintaining blood pressure [24]. Nevertheless, under unpleasant situations, there is a high release of catecholamines into the bloodstream in preparation for the likelihood of faster energy expenditure [24].

According to [52], when an animal is bled out, there is a fall in circulatory pressure, which activates the sympathetic adrenal medullary nervous system, resulting in the release of adrenaline and noradrenaline from the sympathetic endings. Adrenaline and noradrenaline are released from the adrenal medulla to maintain body homeostasis and to regulate several body functions, including maintenance of blood pressure under normal physiological conditions [24].

### 4.3. Influence of Slaughter Positions on EEG Recording

In this study, alpha, beta, delta, theta, Ptot, and F50 increased significantly post-slaughter. Similar results have been reported in goats [53], where EEG activity increased significantly after neck cut compared to before neck cut. Zulkifli et al. [54] found a significant increase in alpha, beta, and F50 after neck cut in cattle. Likewise, Gibson et al. [55] reported an increase in F50 and Ptot in cattle after neck cut. An increase in F50 has been reported to accompany a rise in alpha and beta activity in response to a painful stimulus [55]. Nociception and pain in animals can be determined through analysis of the EEG effects of noxious stimuli [56]. An increase in the root mean square (RMS) for the alpha, beta, delta, and theta waveforms, respectively [53], as well as median frequency (F50) and total power (Ptot) [53], have been correlated with nociception and pain in animals.

In this study, a significant change in the EEG parameters post-slaughter in both the upright and lateral position groups was observed. However, the change in F50 was significantly higher in the upright position than in the lateral position. The current study is the first reported study to examine EEG changes associated with slaughter positions, and thus, comparison with data from similar studies was not possible, representing a major technical challenge. However, a few studies on cattle and goats have reported changes in EEG parameters before and after slaughter [53,54,55]. Moreover, results regarding stress-related hormones can be considered when verifying the aftermath of slaughter positions on the EEG parameters. In this study, adrenaline and noradrenaline hormone levels were seen to be slightly higher in animals slaughtered in vertically upward positions than those in animals slaughtered in the lateral position. This suggests that the EEG parameters were affected by the slaughter positions.

Non-stressed and relaxed states are represented by alpha waves, whereas stress or fear are represented by beta waves; delta waves, meanwhile, represent sleep and unconsciousness. Thus, an increase in alpha and beta waves suggests an element of stress, whereas an increase in delta waves suggests that animals were unconscious after neck cut and an increase in F50 suggests pain in response to cut. In this study, post-slaughter theta activity was higher in animals in the upright position compared to those in the lateral position. One study has reported a transition of EEG from beta waves at baseline to theta waves during examination, representing an inability to cope with examination stress [57]. There is a possibility that animals in the upright position were struggling to maintain their standing positions and experienced failure to cope with the situation, which might have resulted in higher theta waves compared to animals in the lateral position. Thus, higher F50 and delta waves in the upright position compared to those in the lateral position suggest that animals slaughtered in the upright position were affected more than to those in the lateral position.

## 5. Conclusions

The current results from the electroencephalogram showed that, based on MF, total power (Ptot), and delta waves, animals subjected to slaughter in the upright position may experience more stress and pain than animals slaughtered in lateral positions. Animals slaughtered in the upright position showed higher blood sugar levels, lactic acidemia, catecholamines, and liver enzyme activity. These findings suggest that by using the lateral slaughter position, we are able to minimize stress and pain in animals subjected to Halal slaughter when restrained in a modified Mark IV box.

## Figures and Tables

**Table 1:** Differences in blood biochemical parameters in cattle subjected to different slaughter positions before slaughter (T1), and after neck cut (T2).

Parameter	Treatment	Sampling Period		Trt * Period
		T1	T2	
Glucose	Lateral	4.50 ± 0.07 ^ay^	4.78 ± 0.12 ^ay^	
(mmol/L)	Upright	5.17 ± 0.04 ^bx^	5.52 ± 0.05 ^ax^	0.7008
	*p*-value	<0.0001	<0.0001	
Creatine kinase	Lateral	198.90 ± 8.08 ^ay^	229.60 ± 15.7 ^ay^	
(U/L)	Upright	248.77 ± 5.75 ^bx^	275.44 ± 7.76 ^ax^	0.8474
	*p*-value	0.0001	0.0219	
Lactate dehydrogenase	Lateral	1274.60 ± 68.0 ^bx^	1415.00 ± 39.7 ^bx^	
(U/L)	Upright	1556.44 ± 91.29 ^ax^	1728.67 ± 50.93 ^ax^	0.8071
	*p*-value	0.0226	0.0001	
Calcium	Lateral	2.10 ± 0.07 ^ax^	2.20 ± 0.03 ^ax^	
(mmol/L)	Upright	2.18 ± 0.10 ^ax^	2.14 ± 0.05 ^ax^	0.3231
	*p*-value	0.4983	0.4059	
Total Protein	Lateral	75.55 ± 1.95 ^bx^	83.19 ± 1.50 ^ax^	
(g/L)	Upright	78.83 ± 3.35 ^ax^	78.51 ± 3.06 ^ax^	0.1224
	*p*-value	0.3983	0.1745	
Creatinine	Lateral	178.50 ± 7.91 ^ax^	164.80 ± 5.75 ^ax^	
(µmol/L)	Upright	176.66 ± 11.8 ^ax^	186.67 ± 12.80 ^ax^	0.2320
	*p*-value	0.8973	0.1250	
Lactate	Lateral	3.89 ± 0.33 ^ay^	4.74 ± 0.45 ^ax^	
(mmol/L)	Upright	5.22 ± 0.42 ^ax^	5.51 ± 0.11 ^ax^	0.446
	*p*-value	0.0237	0.1353	

^a,b^ Means within the same row with different superscripts are significantly different at *p* < 0.05; ^x,y^ means within the same column with different superscripts are significantly different at *p* < 0.05. Abbreviations: before slaughter (T1), and after neck cut (T2). * denotes interaction.

**Table 2:** Changes in catecholamine parameters in cattle subjected to slaughter positions before slaughter (T1) and after neck cut (T2).

Parameter	Treatment	Sampling Period			
		T1	T2	*p*-Value	Trt * Period
Adrenaline	Lateral	1524.11 ± 7.75 ^by^	2736.47 ± 6.95 ^ay^	<0.0001	<0.0001
(pg/mL)	Upright	1625.87 ± 7.36 ^bx^	3211.56 ± 28.21 ^ax^	<0.0001	
	*p*-value	<0.0001	<0.0001		
Noradrenaline	Lateral	244.85 ± 13.71 ^by^	287.83 ± 1.03 ^ay^	0.0108	0.5070
(pg/mL)	Upright	317.64 ± 7.77 ^bx^	349.61 ± 4.05 ^ax^	0.0045	
	*p*-value	0.0001	<0.0001		

^a,b^ Means within the same row with different superscripts are significantly different at *p* < 0.05. ^x,y^ Means within the same column with different superscripts are significantly different at *p* < 0.05. Abbreviations: before slaughter (T1) and after neck cut (T2). * denotes interaction

**Table 3:** Electroencephalographic changes in cattle subjected to slaughter positions before slaughter (T1) and after neck cut (T2).

Parameter	Treatment	Sampling Period			
		T1	T2	*p*-Value	Trt * Period
Alpha (µv)	Lateral	1.44 ± 0.05 ^bx^	5.03 ± 0.23 ^ax^	<0.0001	0.2085
	Upright	1.25 ± 0.04 ^by^	5.37 ± 0.34 ^ax^	<0.0001	
	*p*-value	0.0059	0.4187		
Beta (µv)	Lateral	2.35 ± 0.07 ^bx^	9.31 ± 0.42 ^ax^	<0.0001	0.2511
	Upright	2.35 ± 0.08 ^bx^	8.59 ± 0.44 ^ax^	<0.0001	
	*p*-value	0.9925	0.2422		
Delta (µv)	Lateral	8.18 ± 0.56 ^bx^	43.41 ± 2.01 ^ay^	<0.0001	0.0265
	Upright	7.35 ± 0.46 ^bx^	50.84 ± 3.03 ^ax^	<0.0001	
	*p*-value	0.2616	0.0419		
Theta (µv)	Lateral	1.81 ± 0.09 ^bx^	7.53 ± 0.43 ^ax^	<0.0001	0.0357
	Upright	1.42 ± 0.04 ^by^	8.72 ± 0.60 ^ax^	<0.0001	
	*p*-value	0.0002	0.1109		
Ptot (µv)	Lateral	12.04 ± 0.46 ^bx^	58.60 ± 2.05 ^ax^	<0.0001	0.082
	Upright	11.42 ± 0.43 ^bx^	63.49 ± 2.32 ^ax^	<0.0001	
	*p*-value	0.3268	0.1158		
MF (µv)	Lateral	12.45 ± 0.38 ^by^	15.06 ± 0.37 ^ay^	<0.0001	0.5478
	Upright	15.27 ± 0.52 ^bx^	18.52 ± 0.74 ^ax^	<0.0001	
	*p*-value	<0.0001	<0.0001		

^a,b^ Means within the same row with different superscripts are significantly different at *p* < 0.05. ^x,y^ Means within the same column with different superscripts are significantly different at *p* < 0.05. Abbreviations: before slaughter (T1) and after neck cut (T2). * denotes interaction

## Data Availability

Not applicable.
